# Supplementary material for: Transcriptome differentiation in Cryptomeria japonica trees with different origins growing in the north and south of Japan
Source: PLoS One. 2025 Sep 26;20(9):e0320549. doi: 10.1371/journal.pone.0320549 (PMC12469258; doi:10.1371/journal.pone.0320549)
Supplement: S1 Fig — PCA was conducted using 19 climatic variables obtained from WorldClim [26] or calculated from current climate data retrieved from AMGSD (denoted by _C) [30]. Source populations are color-coded by genetic group: blue (ura-sugi), yellow (omote-sugi), and red (yaku-sugi). Common garden sites are shown in dark green (growth period) and light green (past climate). (PPTX) [file pone.0320549.s001.pptx]

## Slide 1
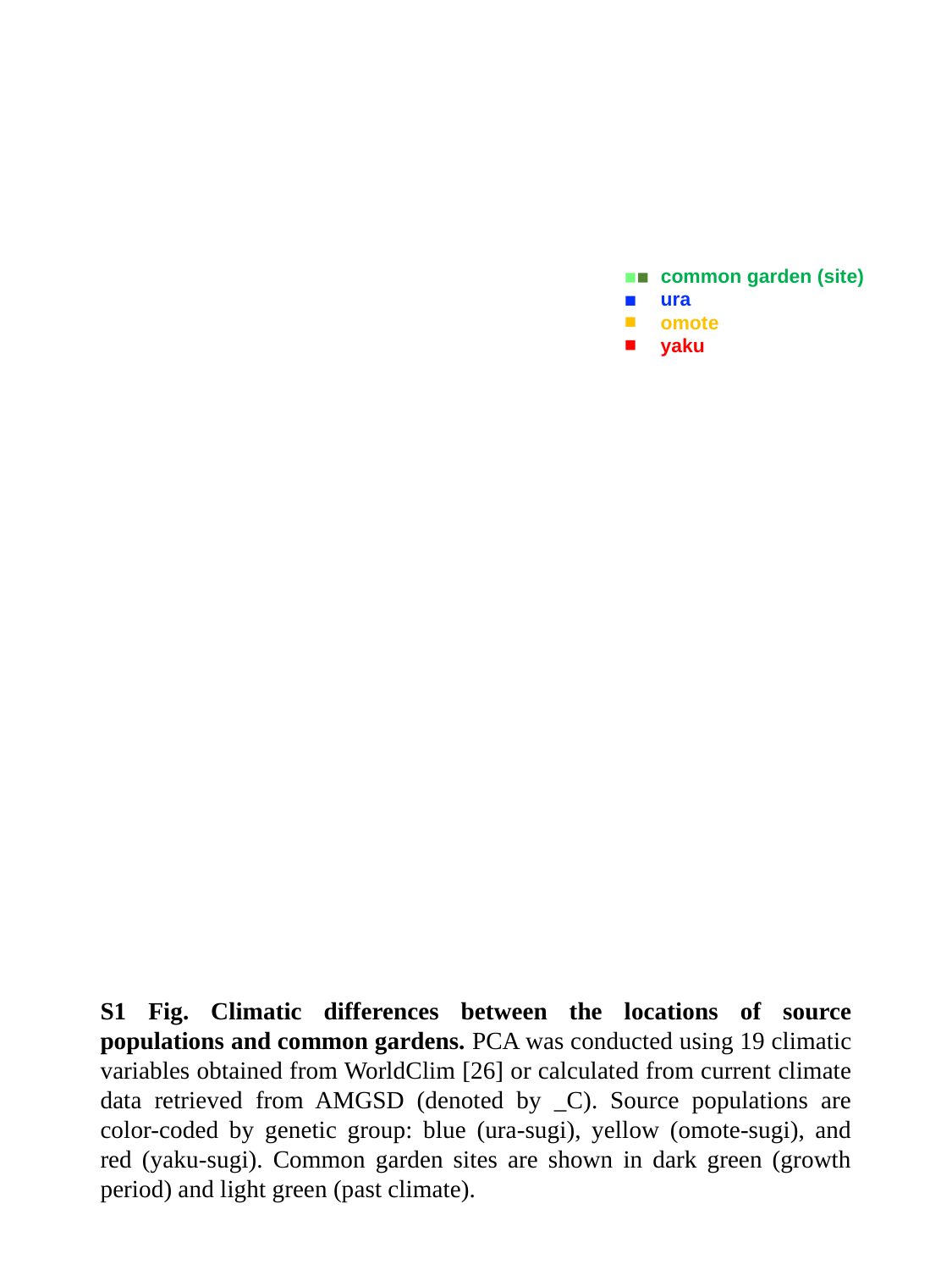

common garden (site)
ura
omote
yaku
S1 Fig. Climatic differences between the locations of source populations and common gardens. PCA was conducted using 19 climatic variables obtained from WorldClim [26] or calculated from current climate data retrieved from AMGSD (denoted by _C). Source populations are color-coded by genetic group: blue (ura-sugi), yellow (omote-sugi), and red (yaku-sugi). Common garden sites are shown in dark green (growth period) and light green (past climate).
