# Supplementary material for: Transcriptome differentiation in Cryptomeria japonica trees with different origins growing in the north and south of Japan
Source: PLoS One. 2025 Sep 26;20(9):e0320549. doi: 10.1371/journal.pone.0320549 (PMC12469258; doi:10.1371/journal.pone.0320549)
Supplement: S2 Fig — A. max air temperature of the day, B total precipitation of the day. Weather data for each common garden obtained from AMGSD [30]. (PPTX) [file pone.0320549.s002.pptx]

## Slide 1
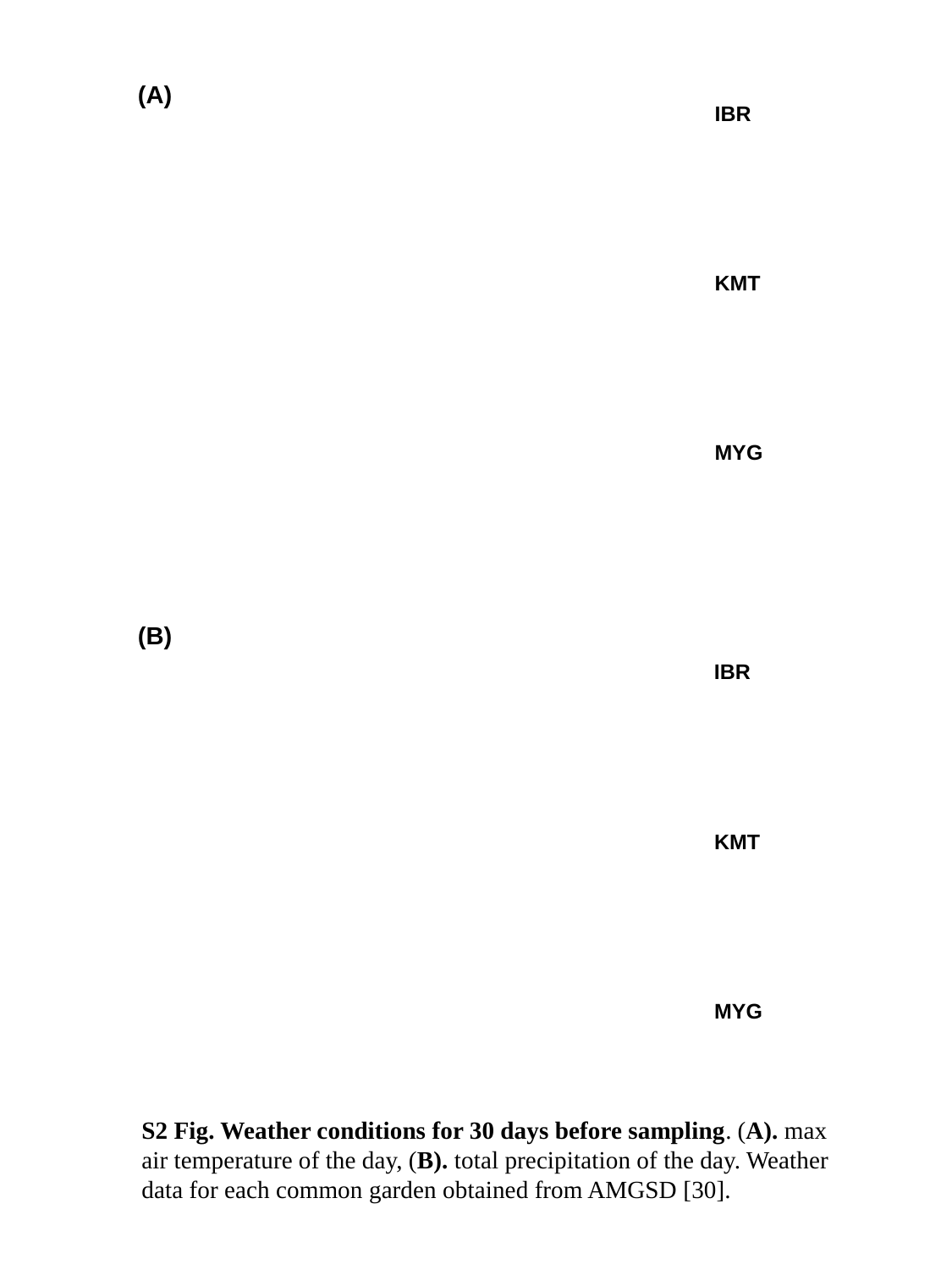

(A)
IBR
KMT
MYG
(B)
IBR
KMT
MYG
S2 Fig. Weather conditions for 30 days before sampling. (A). max air temperature of the day, (B). total precipitation of the day. Weather data for each common garden obtained from AMGSD [30].
