# Supplementary material for: Transcriptome differentiation in Cryptomeria japonica trees with different origins growing in the north and south of Japan
Source: PLoS One. 2025 Sep 26;20(9):e0320549. doi: 10.1371/journal.pone.0320549 (PMC12469258; doi:10.1371/journal.pone.0320549)
Supplement: S3 Fig — Scores of each individual for PC1 and PC2 were calculated using pcadapt [46]. Three genetic groups were shown in blue (ura-sugi), yellow (omote-sugi), and red (yaku-sugi). (PPTX) [file pone.0320549.s003.pptx]

## Slide 1
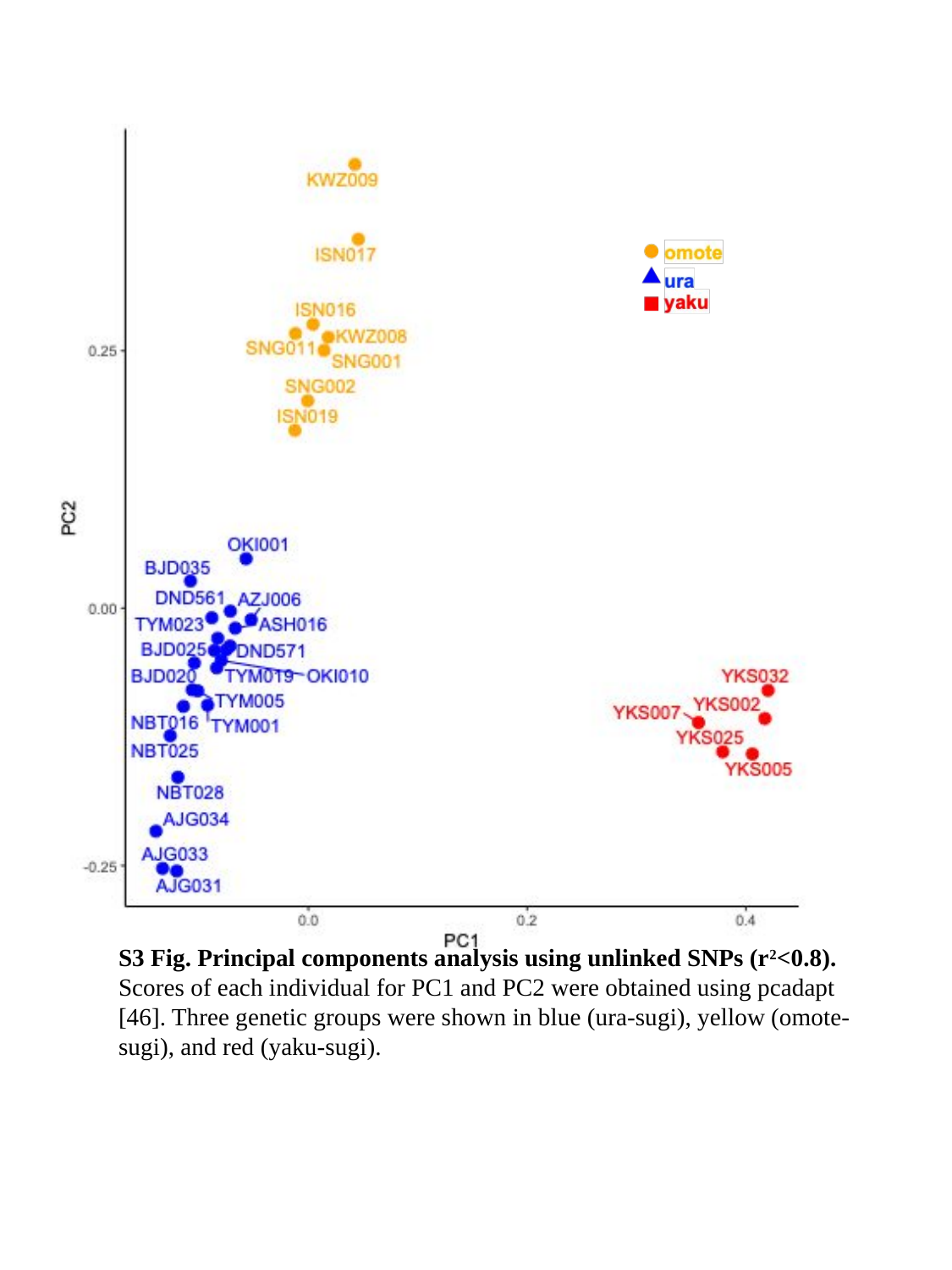

S3 Fig. Principal components analysis using unlinked SNPs (r2<0.8). Scores of each individual for PC1 and PC2 were obtained using pcadapt [46]. Three genetic groups were shown in blue (ura-sugi), yellow (omote-sugi), and red (yaku-sugi).
