# Supplementary material for: Transcriptome differentiation in Cryptomeria japonica trees with different origins growing in the north and south of Japan
Source: PLoS One. 2025 Sep 26;20(9):e0320549. doi: 10.1371/journal.pone.0320549 (PMC12469258; doi:10.1371/journal.pone.0320549)
Supplement: S4 Fig — Darker green indicates higher positive correlations. (PPTX) [file pone.0320549.s004.pptx]

## Slide 1
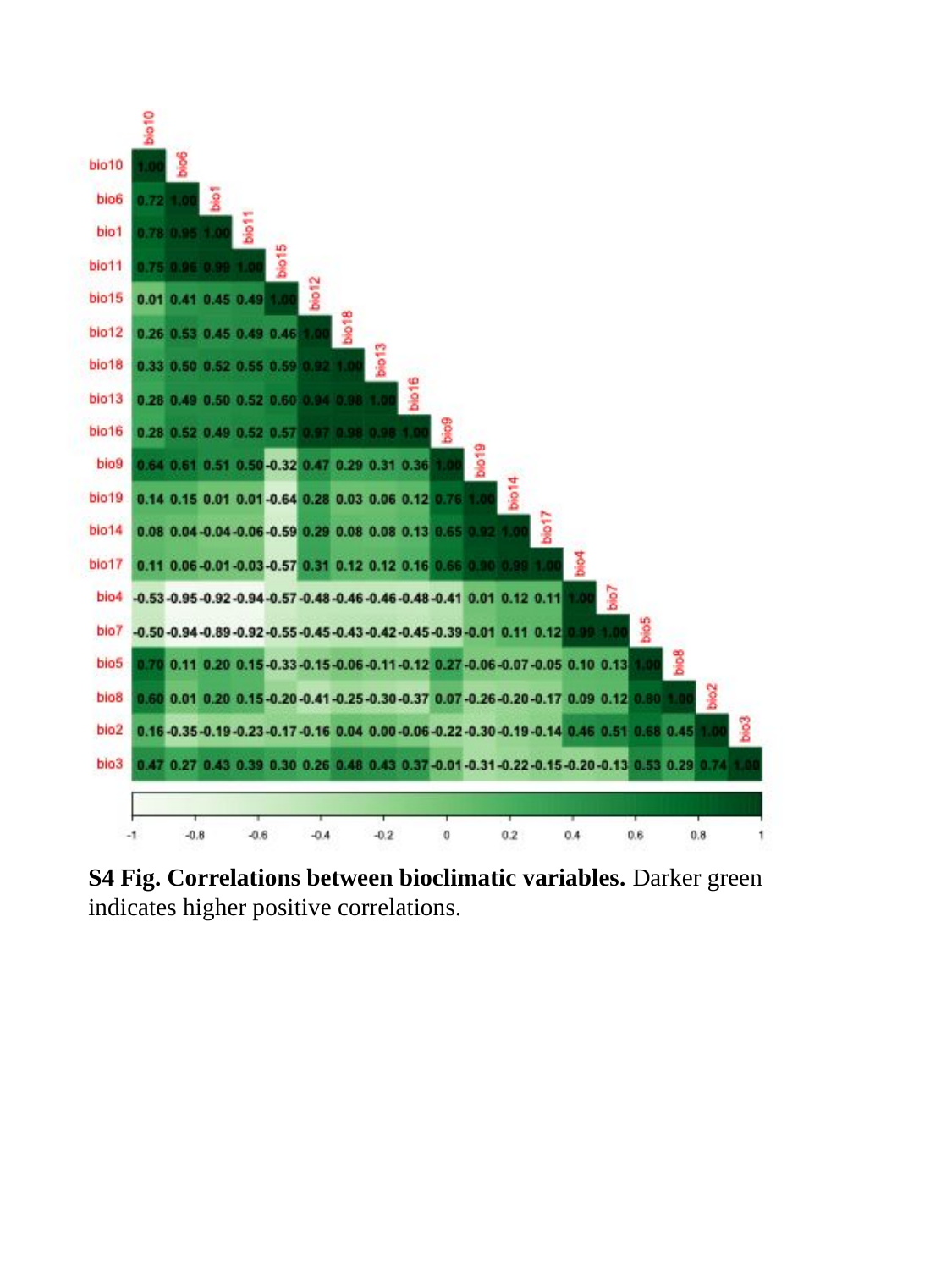

S4 Fig. Correlations between bioclimatic variables. Darker green indicates higher positive correlations.
