# Supplementary material for: Transcriptome differentiation in Cryptomeria japonica trees with different origins growing in the north and south of Japan
Source: PLoS One. 2025 Sep 26;20(9):e0320549. doi: 10.1371/journal.pone.0320549 (PMC12469258; doi:10.1371/journal.pone.0320549)
Supplement: S5 Fig — Correlation was calculated based on variance-stabilized transformation (VST) counts. Darker green indicates a higher correlation. (PPTX) [file pone.0320549.s005.pptx]

## Slide 1
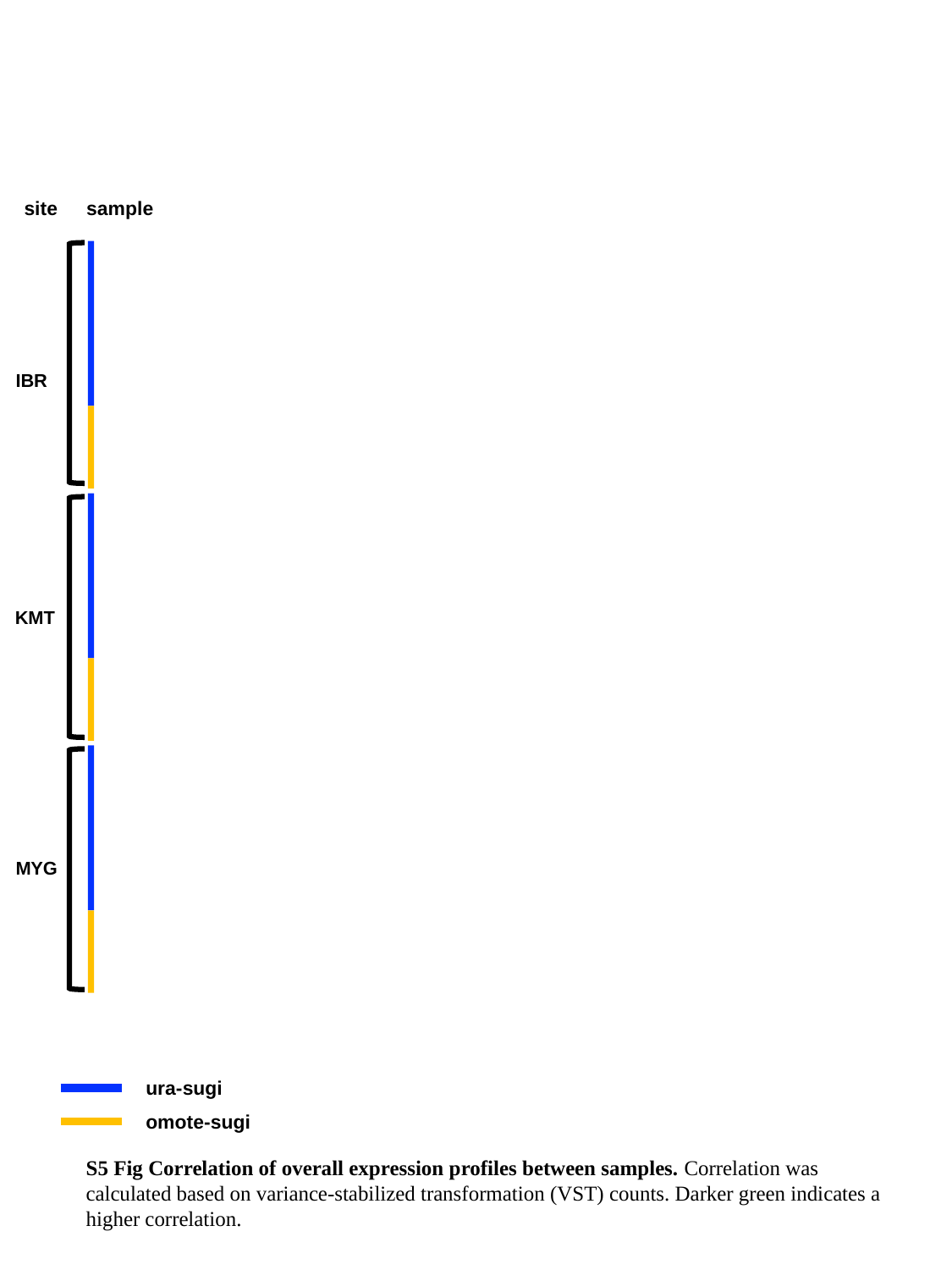

site
sample
IBR
KMT
MYG
ura-sugi
omote-sugi
S5 Fig Correlation of overall expression profiles between samples. Correlation was calculated based on variance-stabilized transformation (VST) counts. Darker green indicates a higher correlation.
