# Supplementary material for: Transcriptome differentiation in Cryptomeria japonica trees with different origins growing in the north and south of Japan
Source: PLoS One. 2025 Sep 26;20(9):e0320549. doi: 10.1371/journal.pone.0320549 (PMC12469258; doi:10.1371/journal.pone.0320549)
Supplement: S6 Fig — A. Predicted exon structures of C. japonica TPS03 transcripts, B. alignment of amino acid sequences of two major transcripts, CJHT.692.8 and CJHT.692.10. In panel A, “Exon start” indicates the start positions of exons based on the C. japonica reference genome, and “Expression” represents the summed TPM values across all samples for each isoform. (PPTX) [file pone.0320549.s006.pptx]

## Slide 1
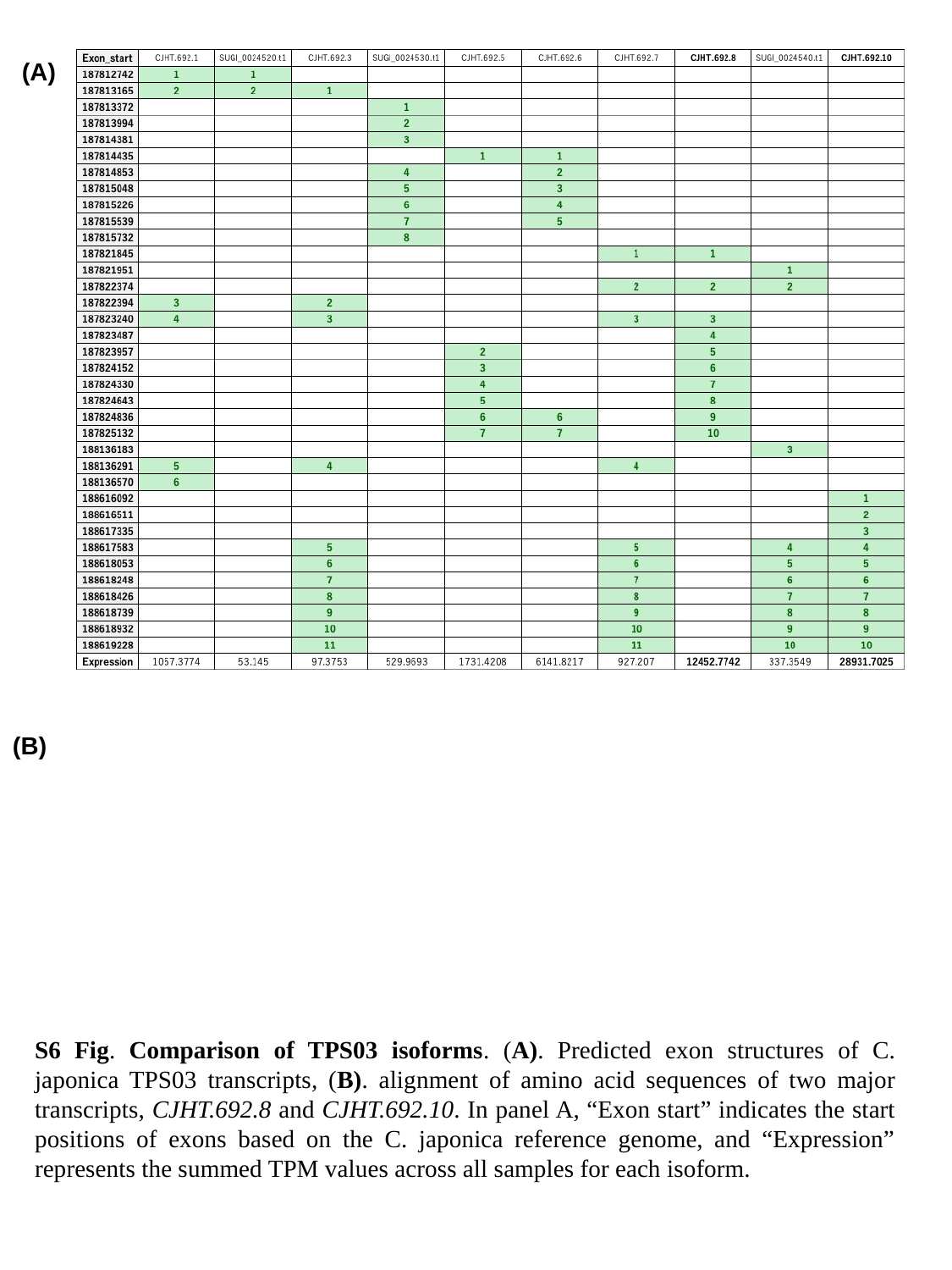

(A)
(B)
S6 Fig. Comparison of TPS03 isoforms. (A). Predicted exon structures of C. japonica TPS03 transcripts, (B). alignment of amino acid sequences of two major transcripts, CJHT.692.8 and CJHT.692.10. In panel A, “Exon start” indicates the start positions of exons based on the C. japonica reference genome, and “Expression” represents the summed TPM values across all samples for each isoform.
