# Supplementary material for: Transcriptome differentiation in Cryptomeria japonica trees with different origins growing in the north and south of Japan
Source: PLoS One. 2025 Sep 26;20(9):e0320549. doi: 10.1371/journal.pone.0320549 (PMC12469258; doi:10.1371/journal.pone.0320549)
Supplement: S7 Fig — A. MEturquoise in KMT and bio6 (mean daily minimum air temperature of the coldest month), B. MEgrey60 in KMT and bio4 (temperature seasonality). Three genetic groups were shown in blue (ura-sugi), yellow (omote-sugi), and red (yaku-sugi). (PPTX) [file pone.0320549.s007.pptx]

## Slide 1
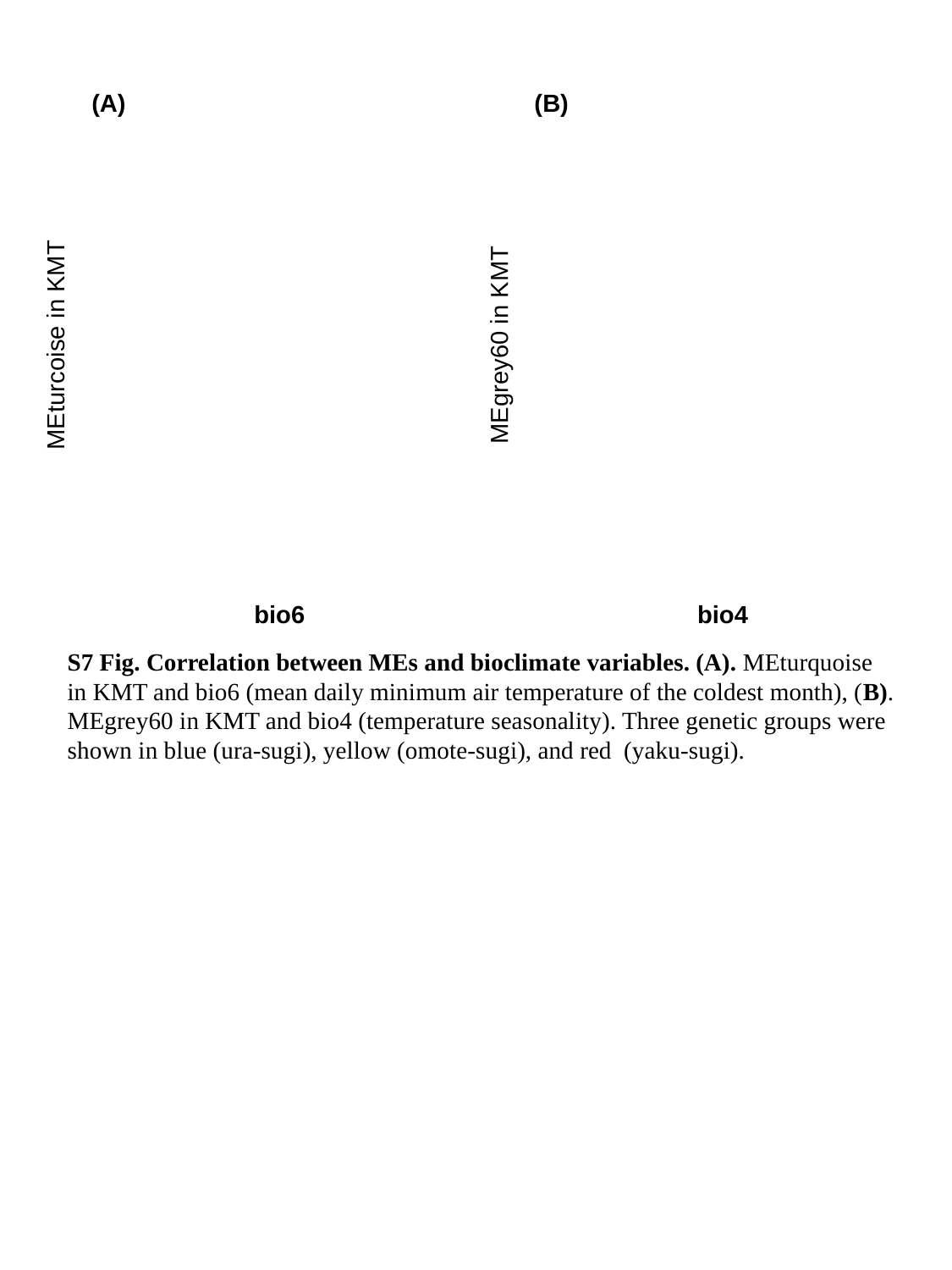

(A)
(B)
MEturcoise in KMT
MEgrey60 in KMT
bio4
bio6
S7 Fig. Correlation between MEs and bioclimate variables. (A). MEturquoise in KMT and bio6 (mean daily minimum air temperature of the coldest month), (B). MEgrey60 in KMT and bio4 (temperature seasonality). Three genetic groups were shown in blue (ura-sugi), yellow (omote-sugi), and red (yaku-sugi).
